# Assessing normative cut points through differential item functioning analysis: An example from the adaptation of the Middlesex Elderly Assessment of Mental State (MEAMS) for use as a cognitive screening test in Turkey

**DOI:** 10.1186/1477-7525-4-18

**Published:** 2006-03-23

**Authors:** Alan Tennant, Ayse A Küçükdeveci, Sehim Kutlay, Atilla H Elhan

**Affiliations:** 1 Academic Unit of Musculoskeletal Disease, University of Leeds, UK; 2 Department of Physical Medicine and Rehabilitation, School of Medicine, University of Ankara, Turkey; 3 Department of Biostatistics, School of Medicine, University of Ankara, Turkey

## Abstract

**Background:**

The Middlesex Elderly Assessment of Mental State (MEAMS) was developed as a screening test to detect cognitive impairment in the elderly. It includes 12 subtests, each having a 'pass score'. A series of tasks were undertaken to adapt the measure for use in the adult population in Turkey and to determine the validity of existing cut points for passing subtests, given the wide range of educational level in the Turkish population. This study focuses on identifying and validating the scoring system of the MEAMS for Turkish adult population.

**Methods:**

After the translation procedure, 350 normal subjects and 158 acquired brain injury patients were assessed by the Turkish version of MEAMS. Initially, appropriate pass scores for the normal population were determined through ANOVA post-hoc tests according to age, gender and education. Rasch analysis was then used to test the internal construct validity of the scale and the validity of the cut points for pass scores on the pooled data by using Differential Item Functioning (DIF) analysis within the framework of the Rasch model.

**Results:**

Data with the initially modified pass scores were analyzed. DIF was found for certain subtests by age and education, but not for gender. Following this, pass scores were further adjusted and data re-fitted to the model. All subtests were found to fit the Rasch model (mean item fit 0.184, SD 0.319; person fit -0.224, SD 0.557) and DIF was then found to be absent. Thus the final pass scores for all subtests were determined.

**Conclusion:**

The MEAMS offers a valid assessment of cognitive state for the adult Turkish population, and the revised cut points accommodate for age and education. Further studies are required to ascertain the validity in different diagnostic groups.

## Background

With the rapid expansion of the population in Turkey, as well as a shift in the population distribution with the emergence of groups susceptible to age-related chronic diseases such as stroke, the need for adequate outcome measures for use in clinical practice becomes paramount. To this end, a programme of adaptation of measures, mostly concerned with activity limitation and quality of life, has been undertaken using standardised adaptation protocols [1-3]. However, there still remains an urgent need for scales measuring aspects of cognitive impairment.

One such scale, the Middlesex Elderly Assessment of Mental State (MEAMS) was developed as a screening test to detect gross impairment of specific cognitive skills in the elderly [4,5]. Thus if problems are identified then more detailed neuropsychological assessment should be undertaken. Clinical psychologists using this scale in the UK suggested to the authors that it would be of use for routine screening in a rehabilitation setting in Turkey, and that it could be used for the adult population, not just for the elderly, given proper adaptation. Thus we set out to adapt the measure for use in Turkey and to assess its internal and external construct validity in an adult population [6], and determine the validity of the existing cut points for passing subtests, given the wide range of the level of education in the Turkish population. This paper focuses on the methodological issues associated with internal construct validity and the cut points, and introduces a novel form of testing the validity of the cut points in these circumstances.

## Methods

### Sample

A sample of 350 normal people aged 16 and over were recruited by one clinical psychologist and two occupational therapists at the Department of Physical Medicine & Rehabilitation, in the School of Medicine of Ankara University (Table 1). The people in this sample were recruited from the hospital staff, relatives of hospital staff and relatives of patients. All participants gave informed consent. Potential subjects for MEAMS administration were questioned regarding their health status and medical history to exclude conditions that might interfere with cognitive performance. These conditions included neurological and psychiatric disorders, including dementia, mental retardation or significant learning disorder, alcoholism, major sight and hearing impairment and the use of psychotropic drugs.

**Table 1:** Distribution of the a) normal subjects and b) patients according to age, gender and educational level, expressed as a % of total within each education/gender group.

**a) Normal subjects**								
Age								
	Primary (n = 132)		Education Middle (n = 97)		High (n = 121)		Total (n = 350)	
	Male	Female	Male	Female	Male	Female	Male	Female
16–30 (n = 84)	21.3	21.2	26.5	22.9	25.4	27.4	24.5	23.6
31–45 (n = 92)	29.8	17.6	30.6	29.2	28.8	27.4	29.7	23.6
46–60 (n = 91)	25.5	22.4	22.4	29.2	25.4	32.3	24.5	27.2
61+ (n = 83)	23.4	38.8	20.4	18.8	20.3	12.9	21.3	25.6
n	47	85	49	48	59	62	155	195

**b) Patients**								
Age								
	Illiterate & Primary (n = 94)		Education Middle (n = 43)		High (n = 21)		Total (n = 158)	
	Male	Female	Male	Female	Male	Female	Male	Female

16–30 (n = 11)	2.1	0.0	21.9	9.1	5.9	25.0	9.3	3.3
31–45 (n = 16)	2.1	6.5	12.5	27.3	23.5	25.0	9.3	11.5
46–60 (n = 42)	27.1	26.1	25.0	27.3	29.4	25.0	26.8	26.2
61+ (n = 89)	68.8	67.4	40.6	36.4	41.2	25.0	54.6	59.0
n	48	46	32	11	17	4	97	61

In addition, because the distribution of normative scores tends to the upper limit, data from 158 consecutive patients with acquired brain injury undergoing rehabilitation were included in the analysis so that the effect of age and education could be viewed across the wider construct of cognition (that is, from those without cognitive impairment to those with severe levels of cognitive impairment). Patients with significant difficulties in language expression or comprehension were excluded, as were those meeting the exclusion criteria applied to the normal population.

### The Middlesex Elderly Assessment of Mental State (MEAMS)

The MEAMS requires the patient to perform a number of simple tasks, each of which is designed to test some aspect of current cognitive functioning. These tasks are grouped into twelve sub-tests each of which has a 'pass score' (Table 2). A screening score of either 0 (fail) or 1 (pass) is assigned to each subtest. These subtests are sensitive to the functioning of different areas of brain, providing separate assessments of perceptual skills, memory, language and executive functions.

**Table 2:** Subtest scores of the original MEAMS.

Subtest	Score range	Pass score	Screening score (Total 0–12)
Orientation	0–5	5	0–1
Name Learning	0–4	2	0–1
Naming	0–3	3	0–1
Comprehension	0–3	3	0–1
Remembering Pictures	0–10	8	0–1
Arithmetic	0–3	3	0–1
Spatial Construction	0–2	2	0–1
Fragmented Letter Perception	0–4	3	0–1
Unusual Views	0–3	2	0–1
Usual Views	0–3	3	0–1
Verbal Fluency	0–10	10	0–1
Motor Perseveration	0–5	3	0–1

Briefly, 'orientation' includes five questions, which test if the patient is orientated in space and time. The patient must answer all five correctly to pass (Table 2). 'Name learning' is for testing memory and asks the patient to remember both the first and second name associated with a photograph given earlier in the test. 'Naming' is a subtest in which three objects are presented to the patient for recognition and naming (e.g. a watch, strap and buckle). Each object correctly identified gains a point. 'Comprehension' requires the subject to name three items from three verbal descriptions. 'Remembering pictures' requires recognition of ten line drawings of common objects, which are presented amongst a set of twenty drawings at a later stage. 'Arithmetic' requires subjects to perform two simple additions and a subtraction. In 'spatial construction' the subject is asked to draw a square and to copy a four-point star. 'Fragmented letter perception' tests the subjects' ability to perceive an item (letters) when it is presented in a fragmented and incomplete form. 'Unusual views' shows objects from unusual angles. Where the subject fails to identify all of these objects, a set of usual views are presented. 'Verbal fluency' involves asking the subject to think of as many animals as possible in two minutes (ten is the pass mark). Finally, 'motor perseveration' tests executive function in five trials. Subsequently a total screening score is calculated as the sum of the screening scores of the 12 subtests.

### Internal construct validity

The internal construct validity (unidimensionality and validity of summed raw score) of the Turkish adaptation of the MEAMS was assessed using the Rasch measurement model [7,8]. The Rasch model is a unidimensional model which asserts that the easier the item the more likely it will be passed, and the more able the person, the more likely they will pass an item compared to a less able person. Formally the probability that a person will affirm an item (in its dichotomous form) is a logistic function of the difference between the person's ability [*θ*] and the difficulty of the item [b] (i.e. the ability required to affirm item i), and only a function of that difference.



where *p*_*ni *_is the probability that person *n *will answer item *i *correctly [or be able to do the task specified by that item],*θ *is person ability, and *b *is the item difficulty parameter. From this, the expected pattern of responses to an item set is determined given the estimated *θ *and b. When the observed response pattern coincides with or does not deviate too much from the expected response pattern then the items constitute a true Rasch scale [9]. Such a scale will be unidimensional and will provide a valid summed score which, through the Rasch transformation, will give objective linear measurement [10]. In the analysis below it is the sum of the twelve subtests which are fitted to the Rasch model. The Rasch model can be extended to cope with items with more than two categories [11], and this involves an explicit 'threshold' parameter (*τ*), where the threshold represents the equal probability point between any two adjacent categories within an item. When subtest scores from the MEAMS were combined (see below) a further derivation for polytomous items, the Partial Credit Model [12] was used:



where no assumptions are made about the equality of threshold locations relative to each item.

### Cut point analysis

Initially, appropriate pass scores for the normal group were examined by distribution scores on each item. Generally all normal respondents would be expected to pass the subtest by scoring the maximum. Thus, where less that 95% scored the maximum, further analysis was undertaken through ANOVA, where evidence was sought of variation by age, gender or education. For these sub-tests, post-hoc tests (Tukey B) identified homogeneous sub groups, showing the influence of the socio-demographic factors. From this analysis, pass rates were selected to reflect significant differences, often varying by age and educational level (gender seemed to be subsumed into education). For educational level 'primary' requires a minimum of 5 years of education; 'middle' 8–11 years (the duration has changed during the lifetime of many of the subjects) and 'Higher education' at least 14 years

Following this initial adjustment, a formal test of the efficacy of the revised cut points for passing a subtest was made through Differential Item Functioning analysis within the framework of the Rasch model [13]. This analysis pooled the data from the normal and patient groups where, in the latter case, the first level of education also included a number of illiterate patients. The basis of the DIF approach lies in the item response logistic function, the proportion of individuals *at the same ability level *who can do a particular task. In the case of cognition, the probability of a person passing a subtest, *at a given level of cognition*, should be the same for younger or older people, men and women, and so on. Thus subtests that do not yield the same response function for two or more groups display DIF. In the case of determining cut points for passing a subtest, as is the case for the MEAMS, a formal test of the validity of the cut point is the absence of DIF. For example, if a cut point is set for passing a subtest and DIF is present for that subtest by age, then further adjustments need to be made to the cut point, adjusting for age, until such a time that DIF is absent. It is crucial to remember that this approach *conditions *on the construct level, in this case cognition. Therefore it does not preclude differences in the distribution of cognitive ability by age, rather states that *at any given level of cognitive ability*, then age should not influence pass rates. RUMM2020 provides both graphical interpretation of DIF, as well as an ANOVA of the residuals. Thus this DIF based ANOVA analysis is subtly different from the distributional analysis of the ANOVA approach which preceded it, as the latter does not condition on the trait. Consequently it is possible to find no significant difference in distribution by groups with the distributional ANOVA, yet find DIF (through the ANOVA of the residuals) when the underlying trait level is taken into account, and vice versa. Where DIF was found, further adjustments to the pass score was made until DIF was found to be absent.

Due to the ceiling effect in the normal population it was necessary to combine some of the subtests for the DIF analysis when comparing invariance between the normal and patient population. This avoided what is called 'extreme' subtests where everyone scored the maximum and which would have precluded their analysis by the Rasch model. Given this, data were then fitted to the Rasch partial credit model to determine overall fit, and how well each subtest fitted the model (to test the validity of summating the 12 subtest pass/fail marks into an overall score). Three overall fit statistics were considered. Two are item-person interaction statistics distributed as a Z statistic with mean of zero and standard deviation of one (which indicates perfect fit to the model). A third is an item-trait interaction statistic reported as a Chi-Square, reflecting the property of invariance across the trait. This means that the hierarchical ordering of the items remains the same at different levels of the underlying trait, indicated by a non-significant Chi-Square. These types of fit statistic are mirrored at the individual item level [14]. First, as residuals (a summation of individual person and item deviations – usually acceptable within the range ± 2.5 and approximately equivalent to the widely reported OUTFIT zsd [15]) and secondly as a chi square statistic (deviation from the model by groups of people defined by their ability level – requiring a non-significant chi square i.e. a *p *value of 0.05 and above, with appropriate adjustment for repeated tests). Misfit of items indicates a lack of the expected probabilistic relationship between the item and other items in the scale. Finally, a measure of reliability, the Person Separation Index (PSI), was computed. This is equivalent to Cronbach's alpha but has the linear transformation from the Rasch model substituted for the ordinal raw score. A value of 0.7 would indicate the ability to differentiate two groups, and 0.8 three groups [16]. Traditionally, values above 0.7 would be adequate for group comparison, above 0.85 for individual use [17].

### Statistical software and significance levels

Rasch analysis was undertaken using the RUMM2020 package [18]. During the Rasch analysis, Bonferroni corrections are applied to both fit and DIF statistics due to the number of tests undertaken [19]. A value of 0.05 is used throughout, and corrected for the number of tests.

## Results

350 subjects were recruited for the normal population, with mean age 45.1 (SD 16.6) (Table 1). 56% were female and 38% had a primary education. 158 patients were also recruited with a mean age of 58.8 (SD 14.7); 38% were female and 43% had a primary education. In addition 16.5% were illiterate.

Initially, scores on the various subtests for the normal group fell into two response groups, the first being those where at least 95% scored the maximum, suggesting that existing pass (cut) scores were appropriate. The second group included those subtests with a wider distribution of scores. Here evidence was sought for variation by age, gender or education. Post-hoc tests (Tukey B) identified homogeneous sub groups, showing the influence of age and education, but not of gender (Table 3). From these analyses, pass rates were modified for six subtest to reflect these significant differences (Post Anova in Table 4a).

**Table 3:** ANOVA post-hoc tests indications of significant differences (non-overlapping homogeneous subsets) in subtests.

Subtest	Age	Education
Orientation	*	*
Name Learning		*
Comprehension	*	*
Arithmetic	*	*
Spatial Construction	*	*
Usual Views	*	*

**Table 4:** MEAMS subtests requiring adjustment of pass scores by ANOVA and Rasch analysis. Bold numbers indicate adjusted scores.

Subtest	Score range	Pass score in original version	Turkish Pass Scores (where P = primary; M = Middle and H = Higher education)											
			Age 16–30			Age 31–45			Age 46–60			Age 61+		
			P	M	H	P	M	H	P	M	H	P	M	H
***a) Post ANOVA***														
Orientation	0–5	5	5	5	5	5	5	5	**4**	5	5	**4**	5	5
Name Learning	0–4	2	2	2	2	2	2	2	**1**	**1**	**1**	**1**	**1**	**1**
Comprehension	0–3	3	3	3	3	3	3	3	3	3	3	**2**	**2**	3
Arithmetic	0–3	3	3	3	3	3	3	3	3	3	3	**2**	3	3
Spatial Construction	0–2	2	**1**	2	2	**1**	2	2	**1**	2	2	**1**	**1**	**1**
Usual Views	0–3	3	3	3	3	3	3	3	**2**	**2**	3	**2**	**2**	3
***b) Post-Rasch***														
Spatial Construction	0–2	2	1	2	2	1	2	2	1	2	2	1	**2**	**2**
Fragmented Letter Perception	0–4	3	**4**	**4**	**4**	**4**	**4**	**4**	3	3	3	3	3	3
Unusual Views	0–3	2	2	2	2	2	2	2	2	2	2	**1**	**1**	**1**

**Figure 1:**
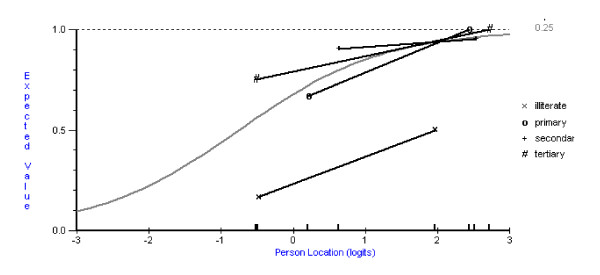
Fragmented Letter Perception subtest DIF by education.

Following this, using the pooled data of both the normal and patient groups, data (based on pass-fail for each subtest) were fitted to the Rasch measurement model. DIF was found for certain subtests by age and education, but not by gender. For example, the 'fragmented Letter Perception' subtest showed clear DIF with the older least educated group having a much lower probability of passing, at any given level of cognitive ability, than all other ages and educational levels (age and education are confounded, that is those who were illiterate were predominately in the oldest age group) (Figure 1). Figure 1 plots this probability with respect to two groups at different levels of cognitive ability, with mean logit scores of around zero for the lower cognitive ability group, and about 2 logits for the higher cognitive ability group (termed class intervals in the Rasch analysis). Following this, pass scores were adjusted (e.g. in the case of 'Fragmented Letter Perception' the pass score was raised for the younger group), and data re-fitted to the model. Three subtests were adjusted in this way (Post-Rasch in Table 4b).

**Figure 2:**
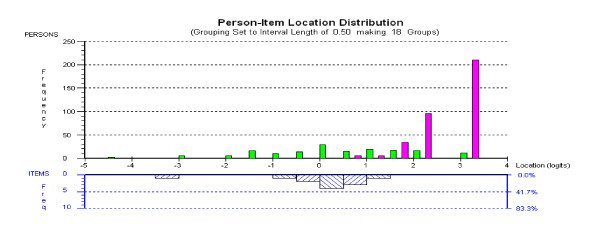
Distribution of the normal (pink) and the patient population (green), and subtest location, on metric cognitive scale.

Fit of the pooled data (normal plus patients) was adequate with all subtests fitting the model. Overall mean item fit was 0.184 (SD 0.319) and person fit was -0.224 (SD 0.557). The item-trait interaction was non-significant, confirming the invariance of items (Chi Sq (df = 8) 19.6, p = 0.012). The Person Separation Index was satisfactory (0.816) indicating the ability of the scale to differentiate at least three groups. Figure 2 shows the clear difference in distribution of the normal (pink) and patient (green) population (at admission), with a mean logit location on the cognitive construct of 2.776 (SD 0.6) for the former, and 0.580 (SD 1.5) for the latter. The final pass scores for all subtests are presented in Table 5 and the percentage passing each subtest are given in Table 6. All subtests significantly discriminated between the normal and patient groups (Chi-Square; p <.001).

**Table 5:** Final pass scores of MEAMS, adjusted for age and educational level for use in Turkey.

Subtest	Score range	Pass score	Turkish Pass Scores (where P = primary; M = Middle and H = Higher education)											
			Age 16–30			Age 31–45			Age 46–60			Age 61+		
			P	M	H	P	M	H	P	M	H	P	M	H
Orientation	0–5	5	5	5	5	5	5	5	4	5	5	4	5	5
Name Learning	0–4	2	2	2	2	2	2	2	1	1	1	1	1	1
Naming	0–3	3	3	3	3	3	3	3	3	3	3	3	3	3
Comprehension	0–3	3	3	3	3	3	3	3	3	3	3	2	2	3
Remembering Pictures	0–10	8	8	8	8	8	8	8	8	8	8	8	8	8
Arithmetic	0–3	3	3	3	3	3	3	3	3	3	3	2	3	3
Spatial Construction	0–2	2	1	2	2	1	2	2	1	2	2	1	2	2
Fragmented Letter Perception	0–4	3	4	4	4	4	4	4	3	3	3	3	3	3
Usual Views	0–3	3	3	3	3	3	3	3	2	2	3	2	2	3
Unusual Views	0–3	2	2	2	2	2	2	2	2	2	2	1	1	1
Verbal Fluency	0–10	10	10	10	10	10	10	10	10	10	10	10	10	10
Motor Preservation	0–5	3	3	3	3	3	3	3	3	3	3	3	3	3

**Table 6:** Percentage pass scores of MEAMS, after adjustment of cut points, by subtest, for each level of educational level and age.

**a) Normative**												
Subtest	**Educational Level **(where P = primary; M = Middle and H = Higher education)											
	Age 16–30			Age 31–45			Age 46–60			Age 61+		
	P	M	H	P	M	H	P	M	H	P	M	H
Orientation	96.4	87.5	100	72.4	100	91.2	83.9	84.0	88.6	84.1	89.5	75.0
Name Learning	92.9	91.7	96.9	86.2	89.7	97.1	67.7	92.0	88.6	79.5	84.2	75.0
Naming	100	100	100	100	100	100	100	100	100	100	100	100
Comprehension	92.9	91.7	96.9	58.6	89.7	97.1	93.5	100	100	95.5	94.7	95.0
Remembering Pictures	100	100	100	100	100	100	96.8	100	100	93.2	100	100
Arithmetic	92.9	100	93.8	82.8	96.6	97.1	90.3	96.0	100	86.4	89.5	100
Spatial Construction	100	100	100	100	86.2	94.1	93.5	88.0	100	93.2	84.2	90.0
Fragmented Letter Perception	100	100	100	100	100	100	100	100	100	100	100	100
Unusual Views	100	100	100	93.1	89.7	100	77.4	80.0	94.3	93.2	89.5	95.0
Usual Views	100	100	100	96.6	89.7	100	90.3	100	100	97.7	89.5	95.0
Verbal Fluency	100	100	100	100	100	100	90.3	100	100	97.7	100	100
Motor Perseveration	100	100	100	100	100	100	100	100	100	95.5	100	100
n	28	24	32	29	29	34	31	25	35	44	19	20

**b) Patients**												
Subtest	**Educational Level **(where P = illiterate/primary; M = Middle and H = Higher education)											
	Age 16–30			Age 31–45			Age 46–60			Age 61+		
	P	M	H	P	M	H	P	M	H	P	M	H

Orientation	100	62.5	50.0	50.0	71.4	60.0	52.0	72.7	66.7	45.2	52.9	37.5
Name Learning	100	50.0	100	50.0	42.9	60.0	52.0	54.5	83.3	40.3	52.9	62.5
Naming	100	100	100	100	100	100	88.0	100	83.3	96.8	100	75.0
Comprehension	100	87.5	100	50.0	28.6	80.0	44.0	90.9	83.3	77.4	100	50.0
Remembering Pictures	100	62.5	100	75.0	57.1	80.0	40.0	81.8	83.3	40.3	70.6	62.5
Arithmetic	100	75.0	50.0	50.0	42.9	100	64.0	90.9	83.3	72.6	88.2	37.5
Spatial Construction	100	50.0	50.0	75.0	14.3	60.0	44.0	45.5	66.7	37.1	29.4	50.0
Fragmented Letter Perception	100	87.5	100	100	85.7	100	76.0	90.9	100	48.4	82.4	75.0
Unusual Views	0.0	75.0	100	75.0	28.6	80.0	44.0	63.6	83.3	29.0	88.2	75.1
Usual Views	100	87.5	100	100	42.9	80.0	52.0	72.7	83.3	38.7	100	75.0
Verbal Fluency	0.0	87.5	100	100	42.9	80.0	54.0	72.7	83.3	74.2	88.2	62.5
Motor Perseveration	100	62.5	100	50.0	57.1	80.0	36.0	63.6	100	27.4	58.8	75.0
n	1	8	2	4	7	5	25	11	6	64	17	8

## Discussion

Introducing cognitive screening questionnaires into a population such as that found in Turkey presents problems over an above those experienced in other countries within Europe or the USA. In the first instance, access to representative samples of the population is difficult, and would require expensive house-to-house visiting. In part, this is necessitated by another factor which has also been shown to influence scores on such measures, that is education level and, in the case of the Turkish population, a substantive minority of illiterate people. This is why we undertook a preliminary study, to try and obtain at least crude estimates of likely normal scores for the Turkish population.

It is possible that, for example, educational levels improve over the years, and that younger people display more skills in some areas that give them an advantage during cognitive testing. This is one reason why normative scores are provided for such tests. Initially we used an ANOVA to identify differences in scores by age, gender and education for the normal group. We took an arbitrary level of 95% passing the original pass score for identifying differences in scores, and only investigated those differences below this level. Given all scores will have a certain level of error, we thought this was a perfectly reasonable starting point for this analysis. The design of the normative population sample meant that group sizes at the level of age and educational group were similar. Although we used parametric ANOVA where perhaps a non-parametric approach would have been more correct, we needed to see the results of post-hoc tests where there were, for example, four groups. Generally only two-way tests are available to determine where pair-wise differences lie in the non-parametric mode.

We also introduced a novel approach by assuming that pass scores should be adjusted to ensure the absence of DIF by age and education on each subtest. Irrespective of distributional aspects associated with age and educational levels, this analysis provides a formal test of the invariance of the subtest (their values determined by the pass score) across groups where bias is expected. Rasch analysis is particularly powerful in that both person and item parameters are estimated independently and thus, for example, item difficulties are estimated independently from the distribution of persons [8]. Normality of distribution is not assumed and thus the lack of normality amongst the patient sample does not affect parameter estimates. Indeed, for purposes of parameter estimation, it is more important to have a good distribution across the trait, which is why we combined the normal and patient population for this purpose. However, we did have to accept some reduced precision when we created combinations of subtests to overcome ceiling effects when we wanted to compare patients with the normal population.

It is important to note that this type of analysis is linked to the internal construct validity of the scale – whether or not it meets the requirements of fundamental measurement – and does not indicate whether or not the pass rate is clinically useful or valid, which is the province of external validity. However, where clinical or other cut points are established by other means, if they fail to meet the requirements of the absence of DIF by relevant groups, then the unidimensionality of any summative score is compromised and group comparison is not valid. Where cut points are already established, as in the current case, we would argue that analysis of DIF represents an elegant mechanism for establishing and correcting for bias under such circumstances. This bias does not necessarily manifest though the ANOVA distributional analysis, and raises important issues about the mechanisms used to adapt scales with cut points into populations which differ from the original.

## Conclusion

In conclusion, the MEAMS demonstrates good internal construct validity for the measurement of mental state in the adult Turkish population, and the revised cut points accommodate for age and educational differences. Although most subtests show a ceiling effect in the normal population, for example, 'naming' and 'fragmented letter perception', no subtest shows a ceiling effect in the patient group, and the subtests have been shown to be highly discriminatory between the normative group and the patients. Further studies are required to ascertain the validity of the instrument in different diagnostic groups. Finally, the use of DIF as a basis for analysing bias in cut points is recommended as a routine assessment where clinical cut points may be confounded by socio-demographic characteristics.

## Competing interests

The author(s) declare that they have no competing interests.

## Authors' contributions

AK, SK and AT were involved with the conception and design of the study. AK and SK arranged the data collection, took part in the interpretation of the data, and the writing of the manuscript. AT and AE undertook the data analysis and interpretation, and also participated in writing the manuscript. All authors read and approved the final manuscript.
